# *O*-Linked *N*-Acetylglucosamine Cycling Regulates Mitotic Spindle Organization*

**DOI:** 10.1074/jbc.M113.470187

**Published:** 2013-08-14

**Authors:** Ee Phie Tan, Sarah Caro, Anish Potnis, Christopher Lanza, Chad Slawson

**Affiliations:** From the ‡ Department of Biochemistry and Molecular Biology,; § KUMC Cancer Center, and; the ¶ Institute for Reproductive Health and Regenerative Medicine, University of Kansas Medical Center, Kansas City, Kansas 64108

**Keywords:** Cell Cycle, Glycosylation, Mitosis, Mitotic Spindle, Phosphorylation

## Abstract

Any defects in the correct formation of the mitotic spindle will lead to chromosomal segregation errors, mitotic arrest, or aneuploidy. We demonstrate that *O*-linked *N*-acetylglucosamine (*O*-GlcNAc), a post-translational modification of serine and threonine residues in nuclear and cytoplasmic proteins, regulates spindle function. In *O*-GlcNAc transferase or *O*-GlcNAcase gain of function cells, the mitotic spindle is incorrectly assembled. Chromosome condensation and centrosome assembly is impaired in these cells. The disruption in spindle architecture is due to a reduction in histone H3 phosphorylation by Aurora kinase B. However, gain of function cells treated with the *O*-GlcNAcase inhibitor Thiamet-G restored the assembly of the spindle and partially rescued histone phosphorylation. Together, these data suggest that the coordinated addition and removal of *O*-GlcNAc, termed *O*-GlcNAc cycling, regulates mitotic spindle organization and provides a potential new perspective on how *O*-GlcNAc regulates cellular events.

## Teaser

**Background:** The addition or removal of the *O*-GlcNAc post-translational modification (*O*-GlcNAc cycling) regulates mitotic progression.

**Results:** Increased *O*-GlcNAc cycling disrupted spindle size and shape partially through the loss of Aurora kinase B phosphorylation of histones. Inhibition of *O*-GlcNAc removal rescued the spindle phenotype.

**Conclusion:** These data suggest that *O*-GlcNAc cycling is a requirement for spindle function.

**Significance:** Proper spindle development requires *O*-GlcNAc cycling.

## Introduction

The segregation of chromosomes into two daughter cells with perfect fidelity is crucial to the process of cell division. Daughter cell segregation is highly dependent upon the construction and organization of the mitotic spindle (1, 2). As a cell enters mitosis, the duplicated chromosomes start to condense and spindle assembly begins. Microtubules radiate outward from the two centrosomes located at opposite poles of the cell and embed themselves into kinetochores assembled on the centromere of each chromosome (3). In this manner, sister chromatids are attached to two microtubules from opposite centrosomes, and when the separation and segregation of these chromosomes ends, each daughter cell will contain an exact copy of all 23 chromosomes (4). Mitotic success rests on the correct orientation of sister chromatids bound to microtubules. Problems at any point in this extensive yet rapid process are detrimental to both subsequent daughter cells. Defects in correct segregation of sister chromatids lead to mitotic catastrophe, apoptosis, or aneuploidy (5).

Mitotic phosphorylation cascades control spindle assembly and organization (6, 7). Initially during prophase, cyclin-dependent kinase 1 (CDK1)^2^ phosphorylates proteins involved in nuclear envelope breakdown and spindle formation. Shortly thereafter, Aurora Kinase A (AurA) and Aurora Kinase B (AurB) phosphorylate the histone H3 variant CENPA, promoting microtubule attachment to kinetochores and chromosome alignment (6). Polo-like kinase 1 (PLK1) phosphorylates targets at the centrosomes, kinetochores, and kinetochore microtubules (8). At the same time, the Chromosomal Passenger Protein Complex (CPC), composed of the proteins AurB, Survivin, Borealin, and the inner centromere protein (INCENP), localize to the spindle midzone to ensure the proper phosphorylation of substrates involved in condensation of chromosomes, correction of microtubule-kinetochore attachments, and the activation of the spindle assembly checkpoint (9–11). As the enzymatic core of the CPC, activation of AurB is of extreme importance during mitosis (12, 13) and altered AurB activity can lead to improper chromosome segregation and cell division (14, 15).

In addition to phosphorylation, the post-translational modification of proteins with *O*-linked *N*-acetylglucosamine (*O*-GlcNAc) regulates cell cycle progression (16, 17). *O*-GlcNAc occurs on serine or threonine residues of nuclear and cytoplasmic proteins consisting of the addition of a single *N*-acetylglucosamine moiety (18). *O*-GlcNAcylation regulates many cellular processes including cell growth and survival (18). Aberrant *O*-GlcNAcylation underlies various pathologies such as type II diabetes, neurodegenerative diseases and cancer (18). Two highly abundant enzymes add or remove *O*-GlcNAc in response to the cellular environment in a process called *O*-GlcNAc cycling. *O*-GlcNAc transferase (OGT) adds *O*-GlcNAc to proteins using the metabolite UDP-GlcNAc (uridine diphospho-*N*-acetylglucosamine) while *O*-GlcNAcase (OGA) is responsible for its removal (18).

Increased expression of OGT leads to mitotic defects and an increased incidence of aneuploidy (16). These results are partially due to alterations in mitotic signaling cascades (19). CDK1 activity is severely reduced in cells with elevated OGT levels due to an increase in the inhibitory phosphorylation of CDK1 mediated by alterations in the expression of upstream kinases and phosphatases (19). Furthermore, OGT and OGA interact with AurB at the midbody, and together, this signaling complex controls the post-translational state of midbody proteins (17). OGT also associates with the mitotic spindle and increased OGT expression hinders the actions of AurB by reducing phosphorylation of histones at the spindle (20). These data demonstrate the vital role of *O*-GlcNAcylation in organizing events in mitosis.

Herein, we address the function of OGT and OGA in regulating the proper construction and activity of the mitotic spindle. We demonstrate that spindle size, shape, and function are disrupted in OGT or OGA gain of function cells. The disruption of spindle architecture was mediated partially through impairment of spindle signaling pathways. CDK1 inhibitory phosphorylation was increased while AurB signaling was dramatically reduced at the spindle midzone as judged by histone H3 serine 10 phosphorylation. Importantly, OGT and OGA gain of function induced this phenotype; however, the OGA inhibitor Thiamet-G (TMG) in the gain of function cells was able to rescue the disrupted spindle phenotype correlated with an increase in histone phosphorylation. Together, these data suggest a novel mechanism where the well-organized cycling of *O*-GlcNAc at the spindle regulates the proper formation and function of this mitotic apparatus.

## MATERIALS AND METHODS

### 

#### 

##### Antibodies

All antibodies were used at a 1:1000 dilution for immunoblotting. pT288 AurA (1:200 for confocal staining, 3079), Survivin (2802), pS7 CENPA (2187), CENPA (2186), Histone H3 (9717), and pS10 histone H3 (1:500 for confocal staining, 9706) antibodies were all purchased from Cell Signaling Technologies. Hec1 (1:200 for confocal staining, ab3613), β-tubulin (1:1000 for confocal staining, ab6046), pT232 AurB (1:200 for confocal staining, ab61074), PLK1 (1:200 for confocal staining, ab17057), pT210 PLK1 (ab39068), pS10 histone H3 (1:5,000 for confocal staining, ab5176), INCENP (ab36453), Borealin (ab70910), CDC2 (ab18), pY15 CDC2 (ab47594), and AurA (ab13824) antibodies were purchased from Abcam. Actin (A2066), AurB (WHOOO9212M3), α-tubulin (1:1000 for confocal staining, T5168), and γ-tubulin (T6557) antibodies were purchased from Sigma. GFP was purchased from Santa Cruz Biotechnology (sc9996). Antibodies for OGT (AL-28, Al-35), OGA (341), and *O*-GlcNAc (110.6) were a gracious gift from the laboratory of Gerald Hart in the Department of Biological Chemistry at the Johns Hopkins University School of Medicine.

##### Cell Culture

HeLa cells were cultured in DMEM (Sigma) supplemented with 10% fetal bovine serum (FBS, Gemini) and 1% penicillin/streptomycin (Invitrogen). Cells were synchronized into M phase using the double thymidine release method, which allows for ∼85–90% of the cells synchronization into M phase (16, 17). Adenoviruses were used as previously described (16, 21). Briefly, infections were performed at first thymidine release with each virus at a multiplicity of infection (MOI) of 100. Cells were treated with 10 μm Thiamet-G (TMG, S.D. Specialty Chemicals) at the time of the second thymidine release. Synchronization of HeLa cells at metaphase was performed by double thymidine block with 2 mm thymidine (16), followed by release and treatment with *S*-trityl-cysteine (Tocris Bioscience) 6 h post-release (22). Metaphase-anaphase cells were then harvested by mitotic shake off 14 h. post-thymidine release. Cells were ∼95% mitotic. Synchronization of HeLa cells at prophase was accomplished by incubation in DMEM media containing nocodazole for 18 h followed by mitotic shake off resulting in ∼95% of the cells in prophase (17). Infection occurred 24 h prior to nocodazole (Sigma) introduction (17).

##### Immunofluorescence Microscopy

Cells cultured on microscope slides were washed with 1× PBS (phosphate-buffered saline, Sigma) and then fixed with 4% Paraformaldehyde (Sigma) for 20 min. The paraformaldehyde was replaced by 1× PBS, and slides were stored at 4 °C. Cells were then permeabilized with 0.1% Triton X-100 (Sigma) in cold PBS for 20 min. Slides were washed with 1× PBS and then blocked with a TBST blocking solution (0.2% azide, 0.2% powdered dry milk, 12% chicken serum, 1% bovine serum albumin, 100 mm glycine, 0.1% Triton X-100 in 500 ml of Tris buffer pH 7.5, all reagents from Sigma except dry milk) for 1 h. Primary antibody in blocking buffer solution was applied overnight. Slides were then washed with TBST and then incubated with fluorescent secondary antibodies (1:1000, rabbit Alexa-fluor 488 nm A11008, mouse Alexa-fluor 488 nm A21202, rabbit Alexa-fluor 568 nm A10042, and mouse Alexa-fluor 568 nm A11004 Invitrogen) for 1 h. DAPI solution (PBS, 0.01% Triton X-100, 0.001% DAPI, Sigma) was applied for 20 min, and slides were then washed and mounted on cover slips using ProLong Gold Anti-fade (Invitrogen) (17).

##### Cell Lysis and Histone Extraction

Cells were lysed in Nonidet P-40 Lysis Buffer (20 mm Tris-HCl, pH 7.4, 150 mm NaCl, 1 mm EDTA, 1 mm DTT, 40 mm GlcNAc, and 1% Nonidet P-40, all reagents from Sigma) on ice for 30 min with occasional vortexing. Cellular debris was pelleted, and the supernatant removed. For histones, the lysed pellet was washed twice with high salt (500 mm NaCl, Sigma) Nonidet P-40 lysis buffer and then resuspended in 0.25 m HCl and vortexed vigorously for 15 min. The sample was pelleted again, and the histone-enriched supernatant was transferred into a new tube. The pellet was subjected to the HCl treatment a second time, and the second supernatant added to the first. Eight volumes of acetone were added to the supernatants, and samples were incubated at −20° overnight. The acetone-precipitated histones were pelleted and washed with 0.1 m HCl in acetone and then washed twice more with acetone alone. Pellets were air-dried and dissolved in a small amount of sterile water (23).

##### Immunoblotting

All electrophoretic separations were performed with 4–15% gradient polyacrylamide gels (Criterion Gels, Bio-Rad). Cell lysates were mixed with protein solubility mix (100 mm Tris, pH 6.8, 10 mm EDTA, 8% SDS, 50% sucrose, 5% β-mercaptoethanol, 0.08% Pyronin-Y, all reagents from Sigma) and separated on gels, followed by transfer to PVDF membrane (Immobilon, Millipore). All antibodies were used at a 1:1000 dilution for immunoblots (16). Blots were developed using HRP-conjugated secondary (anti-rabbit HRP 170–6515 and anti-mouse HRP 170–6516 Bio-Rad, anti-IgM HRP A8786, and anti-IgY HRP A9046 Sigma) and chemiluminescent substrate (HyGlo E2400 Denville Scientific). Blots were stripped in 100 mm glycine (Sigma) pH 2.5 for 1 h, washed, and then treated as before. All Western blots were repeated a minimum of three times using different experimental samples. For densitometry, images were scanned and quantified using ImageJ software (NIH).

##### Spindle Measurements

Measurement of spindle length, width, and angle as well as centrosome distance and HEC distribution was performed using ImageJ software (NIH) from multiple experiments.

##### Statistical Analysis

Statistical significance of the Western blot densitometry was assessed via pairwise comparison with Bonferroni's correction.

## RESULTS

### 

#### 

##### Gain of Function of OGT or OGA Disrupts Spindle Architecture

**FIGURE 1.**
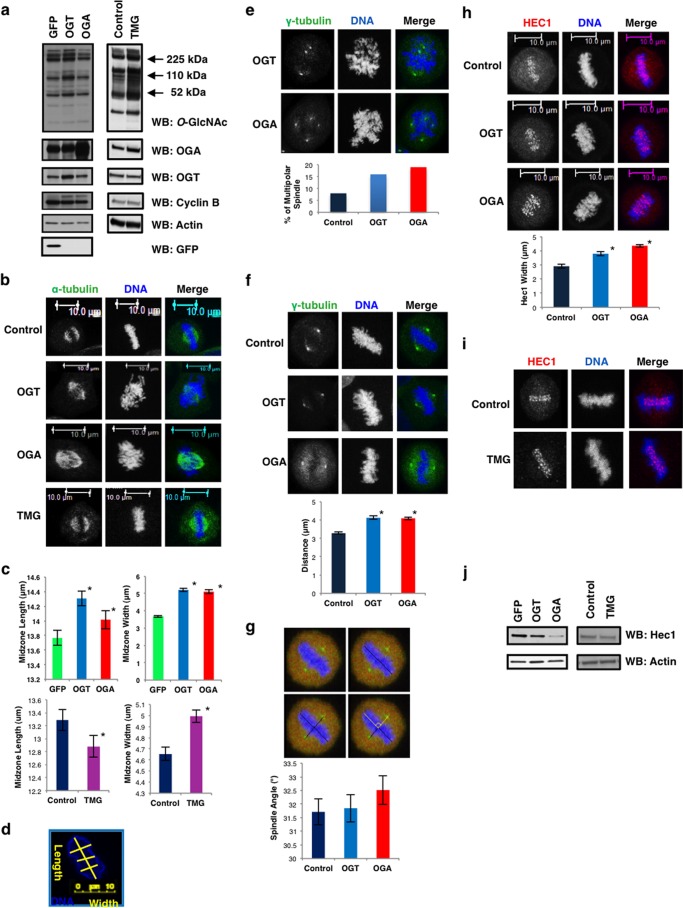
**OGT or OGA gain of function disrupts spindle architecture.** *a*, GFP, OGT, or OGA gain of function or OGA inhibitor Thiamet-G-treated HeLa cells were synchronized to M phase. Western blots were performed for *O*-GlcNAc, OGA, OGT, GFP, cyclin B, and actin. *b–d*, DNA (*blue*) and α-tubulin (*green*) confocal imaging of M phase gain of function OGT/OGA cells, or cells treated with Thiamet-G. Midzone width and length (mean ± S.E., replicate number (n): n*GFP* = 195, *nOGT* = 193, *nOGA* = 198, *nNT* = 195, *nTMG* = 167, *, *p* < 0.005 between GFP/NT *versus* OGT/OGA or TMG) were quantified using ImageJ software as follows: first, the condensed chromatin area color was inverted in order to delineate the edge of the chromatin. Next, the chromatin was measured lengthwise, and the width was measured three times from different sections and averaged. *Yellow lines* are a representative measurement. *e*, multipolar spindles were quantified from confocal images of DNA (*blue*) and γ-tubulin (*green*) M phase synchronized gain of function OGT/OGA cells. *f*, DNA (*blue*) and γ-tubulin (*green*) were confocal imaged at M phase in OGT/OGA gain of function cells. Distance between each centrosome and the midzone was quantified (mean ± S.E., replicate number (n): *nControl* = 93, *nOGT* = 64, *nOGA* = 76, *, *p* < 0.005 between Control *versus* OGT/OGA). *g*, spindle angle measurement schematic in OGT/OGA gain of function cells. The *perpendicular black lines* originating from the centrosomes and the center of the spindle midzone were used to calculate spindle angle shown in *yellow* and plotted as a histogram. *h*, DNA (*blue*) and HEC1 (*red*) were confocal imaged at M phase in OGT/OGA gain of function cells. Average width of HEC1 staining was quantified using ImageJ (mean ± S.E., replicate number (n): *nControl* = 21, *nOGT* = 47, *nOGA* = 50, *, *p* < 0.005 between Control *versus* OGT/OGA). *i*, DNA (*blue*), α-tubulin (*green*), and HEC1 (*red*) were confocal imaged at M phase in TMG-treated cells. *j*, Western blot of HEC1 and actin in OGT/OGA gain of function and TMG-treated cells.

Since elevated expression of OGT or OGA induces mitotic exit defects and aneuploidy (16, 17, 19), we hypothesized that *O*-GlcNAc cycling is important for the formation of the mitotic spindle. We synchronized at M phase OGT/OGA gain of function HeLa cells or control cells expressing GFP. OGT gain of function cells showed an increase in total levels of *O*-GlcNAc while OGA cells lowered *O*-GlcNAc levels (Fig. 1*a*). Synchronized cells were judged mitotic by the expression of Cyclin B with actin as a loading control. Next, we assessed how OGT/OGA gain of function affected spindle chromosome condensation. Spindles from each condition were measured over the course of three separate experiments. Both OGT and OGA gain of function caused an increase in chromosome condensation area (Fig. 1*b*). Condensed chromosome area was significantly larger in cells expressing both OGT and OGA while OGA inhibitor Thiamet-G (TMG) (24) (Fig. 1, *a* and *b*) treatment significantly decreased chromosomal length although width was larger than control (Fig. 1, *c* and *d*). Not only did elevated OGT/OGA expression alter chromosome condensation but we also saw an increase in cells with multipolar spindles (Fig. 1*e*).

Next, we explored the positioning of the centrosome relative to the spindle midzone in cells expressing OGT or OGA. The distance between each centrosome and the midzone was greater in cells with the OGT or OGA gain of function (Fig. 1*f*); however, the spindle angle between the spindle midzone and the two centrosomes was not altered in these cells (Fig. 1*g*) (25). We then measured the organization of the kinetochore. HEC1 (Highly expressed in Cancer) is a component of the Ndc80/HEC1 complex required for kinetochore microtubule attachment to the kinetochore (26). HEC1 staining at the kinetochore is tightly localized to the centromere where the sister chromatids are paired in normal cells (Fig. 1*h*); however, in OGT and OGA gain of function cells the HEC1 staining is more diffuse and less localized to the spindle midzone. The average area of the HEC1 staining is increased in these cells matching the overall increase in condensation width with OGT/OGA gain of function (Fig. 1*h*). HEC1 expression was lower in both OGT and OGA gain of function cells (Fig. 1*j*); although TMG had little effect on HEC1 localization (Fig. 1*i*), expression of HEC1 after TMG treatment was also lower (Fig. 1*j*).

##### OGA Gain of Function Reduces CPC Expression

**FIGURE 2.**
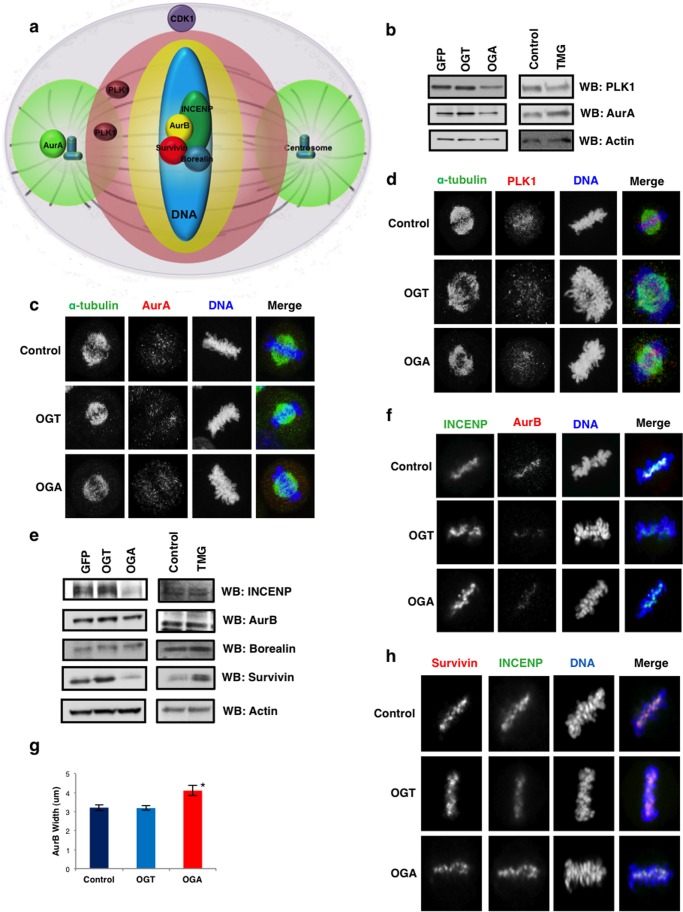
**Disrupted *O*-GlcNAc cycling alters spindle protein expression.** *a* spindle schematic of the locations and area of phosphorylation of mitotic kinases AurA, AurB, and PLK1. *b*, M phase synchronized GFP/OGT/OGA gain of function or TMG-treated cells were Western blotted for PLK1, AurA, and actin. *c*, DNA (*blue*), α-tubulin (*green*), and AurA (*red*) were confocal imaged at M phase in OGT/OGA gain of function cells. *d*, DNA (*blue*), α-tubulin (*green*), and PLK1 (*red*) were confocal imaged at M phase in OGT/OGA gain of function cells. *e*, Western blot of CPC proteins AurB, INCENP, borealin, surviving, and actin as a load control (*f*) DNA (*blue*), INCENP (*green*), and AurB (*red*) were confocal imaged at M phase in OGT/OGA gain of function cells. *g*, AurB width was quantified using ImageJ (mean ± S.E., replicate number (n): *nControl* = 45, *nOGT* = 35, *nOGA* = 43, *, *p* < 0.005 between Control *versus* OGA). *h*, DNA (*blue*), INCENP (*green*), and survivin (*red*) were confocal imaged at M phase in OGT/OGA gain of function cells.

The organization of the spindle is under the control of several mitotic kinases. CDK1 phosphorylates substrates during prophase promoting nuclear envelope breakdown and activation of signaling networks regulated by the kinases PLK1, AurA, and AurB (Fig. 2*a*) (27, 28). In turn, these kinases organize the mitotic spindle by phosphorylating substrates in specific regions of the spindle (Fig. 2*a*) (27, 29). Increased OGT expression leads to increased inhibitory phosphorylation on CDK1 (19) suggesting that *O*-GlcNAcylation is important in the regulation of mitotic signaling events. We first measured whether OGT or OGA gain of function disrupts protein expression of AurA or PLK1. OGA gain of function lowered AurA and PLK1 levels while OGT had little effect (Fig. 2*b*). Previously, we reported that OGT gain of function lowers PLK1 expression slightly at M phase (19); however, those cells were infected at the first thymidine block instead of the first release as in this study. The prolonged OGT overexpression likely promoted the decline in PLK1 expression. TMG treatment lowered PLK1 levels slightly (Fig. 2*b*). The distribution at the spindle of both AurA and PLK1 in OGT/OGA gain of function cells was no different than control (Fig. 2, *c* and *d*). Next, we measured the expression of CPC proteins. OGA cells expressed lower protein levels of INCENP, survivin, and AurB (Fig. 2*e*). OGT cells produced little change while TMG treatment increased survivin protein expression (Fig. 2*e*). Localization of AurB, survivin and INCENP was no different than control; however, AurB staining was more diffuse in the OGA gain of function cells (Fig. 2, *f–h*).

##### Phosphorylation of AurA, AurB, and PLK1 Is Normal in the Gain of Function or TMG-treated Cells

**FIGURE 3.**
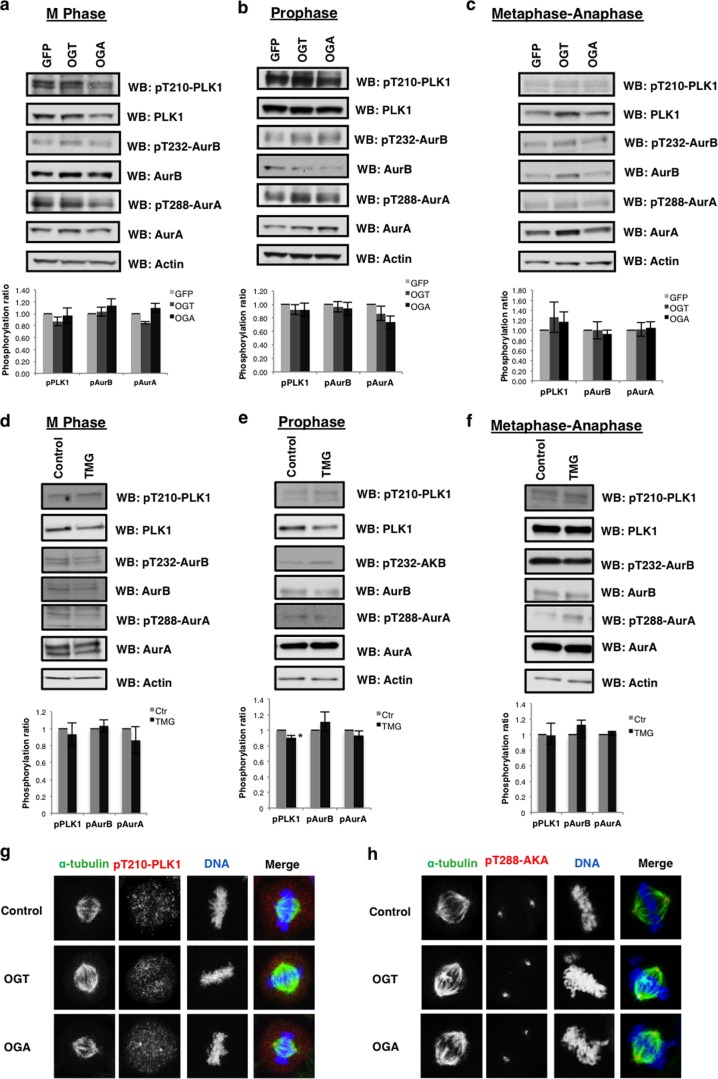
**The effects OGT/OGA gain of function or TMG treatment on mitotic kinase phosphorylation.** *a–c*, GFP, OGT, or OGA gain of function cells were synchronized to (*a*) M phase, (*b*) prophase, (*c*) and metaphase-anaphase. Blots were probed for pT210-PLK1, PLK1, pT232-AurB, AurB, and pT288-AurA, AurA, and actin as a load control. *d–e*, TMG-treated cells were synchronized to (*d*) M phase, (*e*) prophase, (*f*) and metaphase-anaphase. Blots were probed for pT210-PLK1, PLK1, pT232-AurB, AurB, and pT288-AurA, AurA, and actin as a load control. Densitometry of kinase phosphorylation was normalized relative of the kinases and actin to the level then averaged from a minimum of three experiments, *, *p* < 0.005 between GFP/NT *versus* OGT/OGA or TMG. (*g*) DNA (*blue*), α-tubulin (*green*), and pT210-PLK1 (*red*) were confocal imaged at M phase in OGT/OGA gain of function cells. *h*, DNA (*blue*), α-tubulin (*green*), and pT288-AurA (*red*) were confocal imaged at M phase in OGT/OGA gain of function cells.

Phosphorylation of AurA, PLK1, and AurB is required for full enzymatic activation; therefore, we measured whether OGT/OGA gain of function altered the activating phosphorylations on AurA, PLK1, and AurB. Activation of AurA occurs early in prophase and is mediated by AurA interactions with TPX2, Ajuba, and Bora (30–32). This protein complex promotes AurA auto-phosphorylation at threonine 288 (33, 34). In OGT/OGA gain of function cells, AurA phosphorylation was not significantly different from control cells (Fig. 3*a*). Since AurA is active in early prophase, we synchronized cells into prophase by nocodazole treatment. We saw a slight decline in AurA phosphorylation in these cells (Fig. 3*b*). We also measured phosphorylation in cells synchronized at the metaphase-anaphase transition after *S*-trityl-cysteine treatment. In these cells we saw no change in phosphorylation (Fig. 3*c*). Next, we measured the activating threonine 210 phosphorylation on PLK1 (35). We observed no changes in PLK1 phosphorylation from M phase, prophase, or metaphase-anaphase synchronized OGT/OGA gain of function cells (Fig. 3, *a–c*). These data agree with previous reports demonstrating no change in PLK1 phosphorylation in OGT gain of function cells. Finally, we looked at the AurB activating auto-phosphorylation on threonine 232 (36). We saw no difference in AurB phosphorylation under different mitotic synchronization methods (Fig. 3, *a–c*). OGA inhibition had no effect on the activating phosphorylations except for a slight but significant decline in phosphorylated PLK in prophase synchronized cells (Fig. 3, *d–f*). We saw no disruption of localization at the spindle of phosphorylated AurA and PLK1 in OGT/OGA gain of function cells (Fig. 3, *g–h*).

##### CDK1 Inhibitory Phosphorylation Is Altered in OGT/OGA Gain of Function Cells as Well as TMG-treated Cells

**FIGURE 4.**
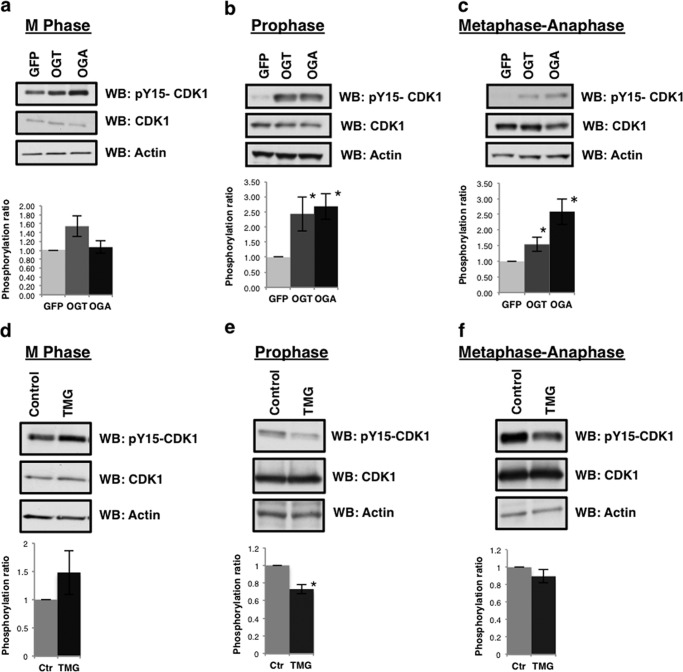
**Altered *O*-GlcNAc cycling increases CDK1 inhibitory phosphorylation.** *a–c*, GFP, OGT, or OGA gain of function cells were synchronized to (*d*) M phase, (*e*) prophase, or (*f*) metaphase-anaphase. Western blots were probed for pY15-CDK1 and CDK1. *d–f*, TMG-treated cells were synchronized to (*d*) M phase, (*e*) prophase, or (*f*) metaphase-anaphase. Western blots from TMG-treated M phase cells were probed for pY15-CDK1 and CDK1. Densitometry of CDK1 phosphorylation was normalized relative to the level of CDK1 and actin then averaged from a minimum of three experiments, *, *p* < 0.005 between GFP/NT *versus* OGT/OGA or TMG.

Previously, we reported an increase in the inhibitory phosphorylation of CDK1 in OGT gain of function cells (19). Again, we synchronized cells either at M phase, prophase, or metaphase-anaphase and probed for CDK1 tyrosine 15 phosphorylation. Similar to previous reports, we continued to see a robust significant increase in tyrosine 15 phosphorylation in OGT gain of function cells; although, OGA gain of function cells did not have consistently elevated tyrosine 15 phosphorylation when only synchronized into M phase. This increase in CDK1 phosphorylation is consistently elevated all prophase and metaphase-anaphase extracts (Fig. 4, *a–c*). TMG-treated cells had higher levels of CDK1 phosphorylation in M phase-synchronized cells although the difference was not statistically significant (Fig. 4, *d–f*); however, prophase synchronized cells had significantly lower CDK1 phosphorylation in the TMG-treated samples while metaphase-anaphase CDK1 phosphorylation was slightly lower compared with control. The difference in the TMG-treated M phase extracts compared with the other synchronization methods is interesting and suggest that cells might be populating all the stages of M phases including telophase when CDK1 phosphorylation would be higher.

##### OGT/OGA Gain of Function Cells Have Significantly Reduced Centromere Protein A Phosphorylation

**FIGURE 5.**
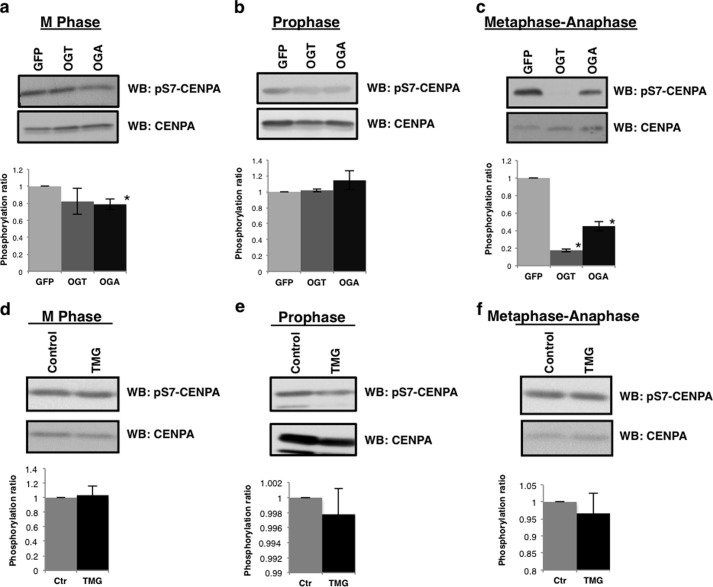
**OGT or OGA gain of function disrupts AurB phosphorylation of CENPA.** *a–c*, GFP, OGT, or OGA gain of function cells were synchronized to (*a*) M phase, (*b*) prophase, (*c*) and metaphase-anaphase. Western blots were probed for pS7-CENPA and total CENPA. *d–f*, TMG treated cells were synchronized to (*d*) M phase, (*e*) prophase, (*f*) and metaphase-anaphase. Western blots were probed for pS7- CENPA and total CENPA. Densitometry of pS7-CENPA was normalized relative to the level of CENPA and averaged from a minimum of three experiments, *, *p* < 0.005 between GFP/NT *versus* OGT/OGA or TMG.

We next looked to measure the activity of AurA and AurB toward spindle substrates in OGT/OGA gain of function cells. Centromere protein A (CENPA) is a histone variant that is phosphorylated at serine 7 by AurA in prophase, which in turn recruits INCENP and AurB to the spindle midzone where AurB phosphorylates CENPA serine 7 during late prophase and metaphase (6, 37). CENPA serine 7 phosphorylation was depressed in mitotically synchronized OGT/OGA gain of function cells (Fig. 5*a*). We synchronized cells in prophase where AurA is the predominate kinase toward CENPA serine 7 and again saw a slight loss of CENPA phosphorylation in the OGT gain of function cells. CENPA phosphorylation was not altered in OGA gain of function cells (Fig. 5*b*). To ascertain the contribution of AurB toward CENPA phosphorylation, we measured phosphorylation in cells synchronized at the metaphase-anaphase transition. Serine 7 phosphorylation was significantly decreased in both OGT/OGA gain of function cells (Fig. 5*c*). CENPA phosphorylation was not changed in cells treated with TMG (Fig. 5, *d–f*).

##### Altered O-GlcNAc Cycling Disturbs Histone H3 Serine 10 Phosphorylation

**FIGURE 6.**
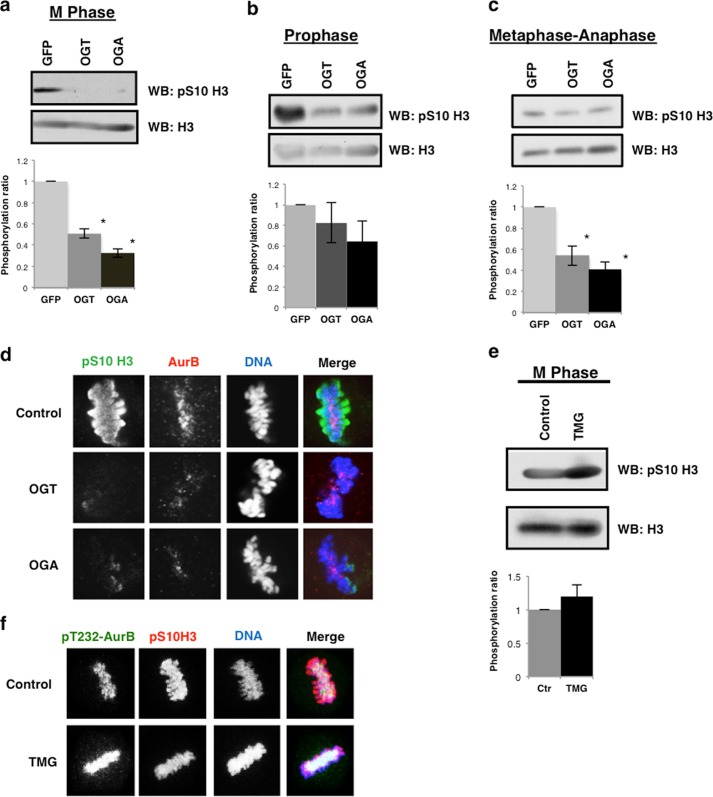
**OGT or OGA gain of function lowers AurB phosphorylation of histone H3.** *a–c*, GFP, OGT, or OGA gain of function cells were synchronized to (*a*) M phase, (*b*) prophase, (*c*) and metaphase-anaphase. Western blots were probed for pS10 H3 and total H3. Western blots were probed for pS10-H3 and total H3. Densitometry of pS10-H3 was normalized relative to the level of H3 and averaged from a minimum of three experiments, *, *p* < 0.005 between GFP *versus* OGT/OGA. (*d*) DNA (*blue*), pS10-H3 (*green*), and AurB (*red*) were confocal imaged at M phase in OGT/OGA gain of function cells. *e*, control or TMG-treated cells were synchronized to M phase and probed for pS10-H3 and total H3. Densitometry of pS10-H3 was normalized relative to the level of H3 and averaged from a minimum of three experiments. *f*, DNA (*blue*), pT232-AurB (*green*), and pS10-H3 (*red*) were confocal imaged at M phase after TMG treatment.

We then measured the AurB-mediated phosphorylation of serine 10 on histone H3, a marker for chromosome condensation (38, 39). Histone H3 is modified by *O*-GlcNAc with the highest levels of H3 *O*-GlcNAcylation occurring during G_1_ phase of the cell cycle (23, 40) while H3 *O*-GlcNAcylation appears lower in M phase (23, 41). Others have reported an increase in *O*-GlcNAcylation at M phase (40). We did not detect any appreciable levels of *O*-GlcNAc on mitotic histones (data not shown). Since H3 serine 10 phosphorylation (H3S10) is reduced in OGT gain of function cells (20). We acid extracted mitotic histones from both OGT and OGA gain of function cells. As previously reported (20), we saw a decrease in H3S10 phosphorylation in OGT gain of function cells at M phase (Fig. 6*a*). OGA gain of function cells had a significant decrease in H3S10 phosphorylation similar to the loss seen in OGT gain of function cells (Fig. 6*a*). In prophase synchronized cells, H3S10 phosphorylation was reduced, although not to the same extent at M phase synchronized cells (Fig. 6*b*). Metaphase-anaphase synchronized cells demonstrated a sharp reduction in H3S10 phosphorylation (Fig. 6*c*). When we visualized H3S10 phosphorylation at the spindle, we saw that staining was reduced at the condensed chromatin with some isolated areas being phosphorylated (Fig. 6*d*). TMG-treated mitotically synchronized cells had slightly higher H3S10 phosphorylation (Fig. 6*e*). H3S10 and Aurora B phosphorylation at the condensed chromosomes was not altered in the TMG-treated cells (Fig. 6*f*). Our results with TMG contradict earlier studies demonstrating that the OGA inhibitor PUGNAc reduced H3S10 phosphorylation (41). We believe this is due to PUGNAc being a less selective OGA inhibitor than TMG and can produce pleotropic effects likely leading to the discrepancy in the H3S10 phosphorylation (42).

##### OGA Inhibition Rescues the Disrupted Spindle Phenotype

**FIGURE 7.**
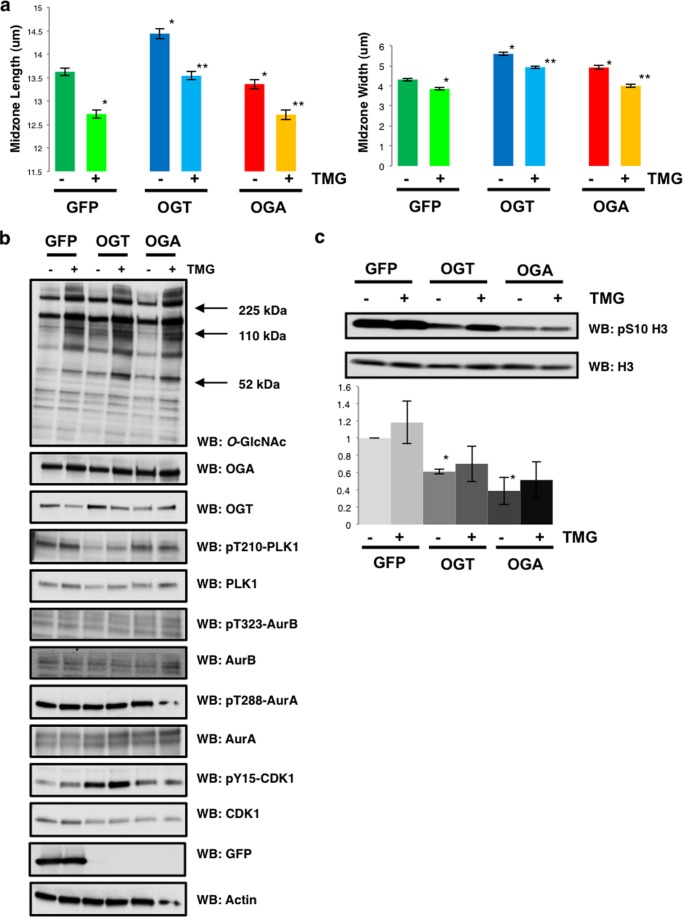
**OGA inhibition rescued disrupted spindle phenotype in OGT/OGA gain of function cells.** GFP, OGT, or OGA gain of function cells were synchronized to M phase and treated with TMG after second thymidine release. *a*, midzone width and length (mean ± S.E., replicate number (n): *nGFP* = 198, *nOGT* = 242, *nOGA* = 178, *nTMG/GFP* = 213, *nTMG/OGT* = 235, *nTMG/OGA* = 223, *, *p* < 0.005 GFP *versus* OGT/OGA, **, *p* < 0.005 GFP/OGT/OGA *versus* TMG-GFP/TMG-OGT/TMG-OGA) were quantified using ImageJ software from confocal images stained for DNA and α-tubulin. *b*, Western blots were probed for *O*-GlcNAc, OGT, OGA, pT210 PLK1, PLK1, pT323-AurB, AurB, pT288-AurA, AurA, pY15-CDK1, CDK1, GFP, and actin as a load control. *c*, Western blots were probed for pS10 and total H3. Densitometry of H3 phosphorylation was normalized relative to the level of H3 and averaged from a minimum of three experiments, *, *p* < 0.005 between GFP *versus* OGT/OGA.

OGT and OGA gain of function consistently gave rise to disrupted spindle phenotypes. Contrary to OGT/OGA gain of function cells, TMG-treated cells had smaller spindles and higher H3S10 phosphorylation. We investigated this apparent discrepancy in spindle size between chemical inhibition of *O*-GlcNAc removal and overexpression of the *O*-GlcNAc cycling enzymes. OGT/OGA gain of function or GFP control HeLa cells were synchronized into M phase via the double thymidine block method. We added TMG to these cells at the time of the G_1_/S release. The cells were fixed, stained for tubulin and DNA 10 h postsecond thymidine release, and chromosome condensation was measured. We measured spindles for each condition over the course of three separate experiments. Condensed chromosome area from gain of function OGT/OGA cells was considerably larger than GFP control cells (Fig. 7*a*). TMG treatment caused a significant reduction in condensation area in GFP control cells; surprisingly, TMG reversed the chromosome condensation errors in both the OGT/OGA gain of function cells (Fig. 7*a*). We then determined if TMG treatment combined with OGT/OGA gain of function changed the phosphorylation of AurA, AurB, PLK1, and CDK1. We saw little difference in the phosphorylation status of these kinases (Fig. 7*b*). Isolated histones from OGT/OGA gain of function cells had significantly lower H3S10 phosphorylation. Interestingly, treatment with TMG produced a trend of partially restored H3S10 phosphorylation that did not, however, achieve statistical significance (Fig. 7*c*).

## DISCUSSION

Disruption in *O*-GlcNAc levels impair M phase progression, alter mitotic signaling cascades, changes the distribution of chromatin marks, and promotes aneuploidy (16, 17, 19, 20). We show that either an OGT or OGA gain of function impairs the correct assembly of the mitotic spindle. Centrosome localization, kinetochore organization, and chromosome condensation are all disrupted, which in turn promotes an increase in multipolar spindles through impaired cytokinesis. The localization and phosphorylation of the mitotic kinases AurA, AurB, and PLK1 is not altered, but both CENPA and histone H3 phosphorylation is lower. Contrary to the phenotype of OGT/OGA gain of function cells, the OGA inhibitor TMG produces more condensed chromosomes, and rescued the disorganized spindle phenotype seen in OGT/OGA gain of function cells.

Previous work supports the fact that OGT and OGA gain of function cause a variety of spindle defects. OGT gain of function causes cytokinesis errors resulting in an increase in polyploidy; furthermore, this is accompanied by a large increase in chromosomal bridges suggesting segregation defects (16, 20). The effects of OGT/OGA gain of function on the spindle are likely through multiple mechanisms since many cellular processes control spindle size and shape such as histone phosphorylation, tubulin polymerization, and condensin complex function (38, 39, 43). OGA inhibitor TMG did reduce spindle length although spindle width was wider than control cells. This data suggest that blocking the turnover of *O*-GlcNAc at the spindle with TMG induces structural changes to the spindle. Later when TMG was used in the gain of function cells both DNA width and length was decreased in all samples suggesting that blocking *O*-GlcNAc cycling will lead to a more condensed spindle phenotype. The alterations in *O*-GlcNAc cycling rate might be interfering with phosphorylation networks. For example, OGT gain of function significantly alters the phosphorylation on at least one hundred mitotic proteins (19). Likely, disruption in *O*-GlcNAc cycling has some influence on the mitotic phosphorylation cascades that control spindle formation such as CDK1 signaling.

Next, we explored the possibility that disrupted *O*-GlcNAc cycling was interfering with mitotic phosphorylation cascades. Our data point to an indirect relationship between the mitotic kinases AurA, AurB, and PLK1 with OGT/OGA. Alteration of *O*-GlcNAc cycling either through gain of function or chemical inhibition did not significantly alter the expression or phosphorylation of AurA, AurB, and PLK1. The expression of INCENP and survivin was lower in the OGA gain of function cells but both still localized to the spindle. Importantly, only a substoichiometric amount of INCENP is needed to catalyze AurB autophosphorylation suggesting that the amount of INCENP at the spindle in OGA gain of function cells is sufficient to activate AurB (36). On the other hand, survivin protein levels were modestly higher when *O*-GlcNAc levels were elevated. The increased amount of survivin is due either to prolonged stability of the protein or a transcription/translation increase. Together, these data suggest that the CPC is still able to organize and promote AurB auto-phosphorylation as normal when *O*-GlcNAc cycling is disrupted. However, substrates of the CPC such as serine 10 on histone H3 were not efficiently phosphorylated in OGT/OGA gain of function cells.

Increased OGT expression increases CDK1 inhibitory phosphorylations at threonine 14 and tyrosine 15 (19). We demonstrate that OGA gain of function increases tyrosine 15 phosphorylation when synchronize to either prophase or metaphase-anaphase. Both OGT and OGA are causing similar effects on CDK1 phosphorylation suggesting that increased expression of OGT/OGA alter the rate of cycling, and this cycling rate is critical for the control of CDK1 phosphorylation. When we incubated cells with TMG, we saw a decrease at prophase while in mitotic and metaphase-anaphase extracts TMG appeared to have no effect. CDK1 phosphorylation is an exceedingly complex process. Multisite phosphorylations on CDK1 promote ultrasensitivity, the process in which increases in phosphorylation induce a threshold input resulting in a switch like change in function (44). The kinase-phosphatase system of Wee1 and Cdc25 control the phosphorylation of CDK1 but CDK1 activation leads to the downstream phosphorylation of these proteins and their inverse activation (Wee1 inhibition and Cdc25 activation) leading to the switch like activation of CDK1 at M phase (44). The Wee1-Cdc25 system can be restored by the actions of the phosphatase PP2A (45, 46); however, at M phase CDK1 activity creates a feed-forward loop inactivating PP2A through phosphorylation (44). Likely, *O*-GlcNAc cycling is affecting not only the Wee1-Cdc25 loop (19) but also the PP2A system. Cdc25 mRNA levels are lower in OGT gain of function cells and MYT1 kinase expression which also controls CDK1 inhibitory phosphorylation is elevated in these cells (19). Additional control of the CDK1 switch by *O*-GlcNAc cycling would provide cells the ability to integrate changes in *O*-GlcNAc levels due to environmental signals into CDK1 activation and establish an even more sensitive control of CDK1 activation.

The effect the changes in *O*-GlcNAc cycling has on mitotic histone phosphorylation is likely not due to *O*-GlcNAcylation of histones. Indeed, histones are modified by *O*-GlcNAc and several sites have been mapped on H2A, H2B, and H4 (23, 47). Histone H3 is also *O*-GlcNAc modified although the site has not been mapped by mass spectrometry (23, 41). *O*-GlcNAcylation of histones is lower at M phase (23). Zhang *et al.* argue through mutational studies that serine 10 is the site of *O*-GlcNAcylation since a histone H3 mutant consisting of a serine 10 to alanine mutation was not efficiently modified by *O*-GlcNAc; however, this does not definitively argue for serine 10 being the *O*-GlcNAc site but rather the serine to alanine mutation impairs OGT activity toward histone H3 (40). Our data does not support serine 10 being *O*-GlcNAcylated in mitotic cells. Should H3S10 be *O*-GlcNAcylated at M phase, we would anticipate increased H3S10 *O*-GlcNAcylation in OGT gain of function cells or after TMG treatment but we could not measure H3S10 *O*-GlcNAcylation after elevation of *O*-GlcNAc levels. Furthermore, several groups detected less *O*-GlcNAc on H3 during M phase suggesting that in mitotic cells serine 10 is not the main site of H3 *O*-GlcNAcylation (23, 41).

**FIGURE 8.**
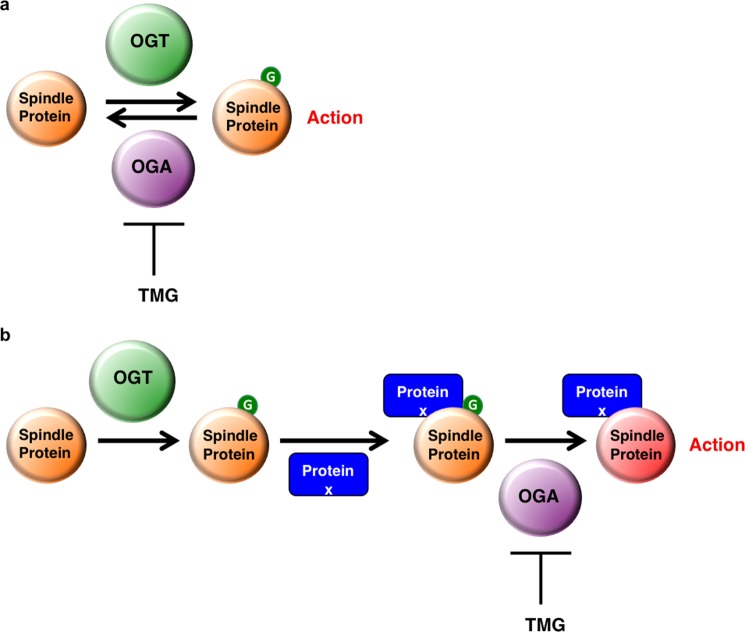
***O*-GlcNAc cycling regulates spindle function.** *a*, schematic of *O*-GlcNAc cycling functioning as an on/off switch with addition of the *O*-GlcNAc residue (*green*) to a protein having direct effect on protein activity. *b*, schematic of *O*-GlcNAc cycling acting as a state machine in which the addition of the *O*-GlcNAc acts as a priming stage allowing for other spindle proteins (Protein X, *blue*) to interact with the *O*-GlcNAc-modified spindle protein. Full activation is only achieved when *O*-GlcNAc is removed.

The reduction of H3S10 phosphorylation in OGT and OGA gain of function cells and the partial rescue of H3S10 phosphorylation by TMG suggest that we need to reexamine conceptually how *O*-GlcNAcylation regulates protein function. In many cases, the addition of the *O*-GlcNAc residue to a protein will have a direct effect on protein activity (Fig. 8*a*). For example, *O*-GlcNAcylation of calcium/calmodulin-dependent protein kinase IV blocks an activating phosphorylation site thereby reducing enzymatic activity (48) while *O*-GlcNAcylation of phosphofructokinase 1 blocks an allosteric regulation site and also reduces enzymatic activity (49). However, our data suggest a model that *O*-GlcNAcylation can prime a protein for a potential physical interaction with other proteins but full activation cannot be achieved until the *O*-GlcNAc is removed which in turn leads to full activation of the protein complex (Fig. 8*b*).

Both OGT and OGA gain of functions cells have a similar disorganized spindle phenotype while TMG treatment partially rescued this phenotype. Three possible mechanisms could explain these actions: 1) The rate of *O*-GlcNAc removal is faster in gain of function cells leading to a disruption of protein-protein interactions facilitated by the *O*-GlcNAc modification, 2) The overexpressed proteins are interacting with and disrupting other protein-protein interactions, or 3) TMG is causing off-target effects. If mechanism two is correct, then TMG treatment in the gain of function cells would not have any effect on the organization of the spindle since *O*-GlcNAc levels are secondary to the increase in OGT/OGA protein expression; however, TMG restores spindle organization and H3S10 phosphorylation arguing for mechanism one. TMG is a chemical inhibitor and could potentially have off-target effects that we cannot completely rule out, but TMG has been demonstrated to be highly specific inhibitor of OGA and induces different cellular responses compared with previous generation OGA inhibitors like PUGNAc (24, 50).

Therefore, we propose a new model mechanism in which OGT *O*-GlcNAcylates proteins localized at the spindle midzone. As OGA is found in a complex with OGT (17, 51), OGA is therefore targeted to the spindle midzone by OGT. The *O*-GlcNAcylated spindle proteins facilitate the interaction of the CPC with spindle substrates (17). Next, OGA removes the modification, and maximal activity of the CPC toward H3S10 occurs mediating a physiological response (Fig. 8*b*). Increased OGT or OGA expression would accelerate *O*-GlcNAc cycling and impair targeting of the CPC to substrates. TMG would elevate *O*-GlcNAcylation and promote the recruitment of the CPC toward spindle substrates like H3S10. TMG would also reduce OGA activity providing enough time for the CPC to properly organize at the spindle midzone and phosphorylate H3S10. However, prolonged inhibition of OGA could be deleterious as well by stabilizing transient interactions or impairing the antagonizing actions of phosphatases or other proteins.

In conclusion the cycling of *O*-GlcNAc is a critical requirement for proper spindle organization. Alteration in the rate of *O*-GlcNAc cycling causes disorganized spindles, increases centrosome-spindle midzone distance, disrupts kinetochores, and causes an increase in multi-polar spindles. A potential contributing factor to the disorganized spindles is the reduction in AurB activity toward histone H3. OGA inhibition can reverse the phenotypes caused by an increase in *O*-GlcNAc cycling, and partially restore AurB activity toward H3. Our data suggest a novel mechanism by which *O*-GlcNAc cycling regulates the function of protein complexes. Furthermore, our data suggest a potentially new model of the role of *O*-GlcNAc cycling in regulating biological processes.

## Footnotes

2 The abbreviations used are:

CDK1: cyclin-dependent kinase 1
PLK: Polo-like kinase
CPC: chromosomal passenger protein complex
INCENP: inner centromere protein
*O*-GlcNAc: *O*-linked *N*-acetylglucosamine
OGA: *O*-GlcNAcase
UDP-GlcNAc: uridine diphospho-*N*-acetylglucosamine
OGT: *O*-GlcNAc transferase
TMG: Thiamet-G
CENPA: centromere protein A.
